# The Moo’D Study: protocol for a randomised controlled trial of A2 beta-casein only versus conventional dairy products in women with low mood

**DOI:** 10.1186/s13063-021-05812-6

**Published:** 2021-12-11

**Authors:** Meghan Hockey, Hajara Aslam, Michael Berk, Julie A. Pasco, Anu Ruusunen, Mohammadreza Mohebbi, Helen Macpherson, Mary Lou Chatterton, Wolfgang Marx, Adrienne O’Neil, Tetyana Rocks, Amelia J. McGuinness, Lauren M. Young, Felice N. Jacka

**Affiliations:** 1 The Food & Mood Centre, IMPACT (the Institute for Mental and Physical Health and Clinical Translation), School of Medicine, Barwon Health, Deakin University, Geelong, Australia; 2 Orygen, The National Centre of Excellence in Youth Mental Health, Centre for Youth Mental Health, Florey Institute for Neuroscience and Mental Health and the Department of Psychiatry, The University of Melbourne, Melbourne, Australia; 3 IMPACT (the Institute for Mental and Physical Health and Clinical Translation), School of Medicine, Barwon Health, Deakin University, Geelong, Australia; 4 Department of Epidemiology and Preventive Medicine, Monash University, Prahran, VIC Australia; 5 Department of Medicine-Western Health, The University of Melbourne, St Albans, VIC Australia; 6 Institute of Public Health and Clinical Nutrition, University of Eastern Finland, Kuopio, Finland; 7 Department of Psychiatry, Kuopio University Hospital, Kuopio, Finland; 8 Biostatistics Unit, Faculty of Health, Deakin University, Burwood, VIC 3125 Australia; 9 Institute for Physical Activity and Nutrition, Faculty of Health, Deakin University, Burwood, VIC 3125 Australia; 10 Institute for Health Transformation, Faculty of Health, Deakin University, Geelong, Australia

**Keywords:** Beta-casein, Milk, Dairy, Psychological distress, Inflammation, Gut microbiota, Depression, Psychiatry, Mental disorders, Randomised controlled trial

## Abstract

**Background:**

Beta-casein is a major protein in cow’s milk, of which A1 and A2 are the most frequent variants. Recent evidence implicates A1 beta-casein consumption in mechanisms that are of potential importance to mental health, yet its possible effects on psychological endpoints remains unknown. The primary aim of the study is to evaluate the comparative effects of consumption of dairy products containing A2 beta-casein versus conventional dairy (i.e. containing both A1 and A2 beta-casein) on symptoms of psychological distress in women with low mood.

**Methods:**

‘The Moo’D Study’ is a 16-week, superiority, 1:1 parallel group, triple-blinded, randomised controlled trial. Ninety women with low mood (Patient Health Questionnaire score ≥ 5) will be randomised to consume either A2 beta-casein only or conventional dairy products. The primary outcome, symptoms of psychological distress, will be measured by the 21-item Depression, Anxiety and Stress Scale. Secondary outcomes will include symptoms of depression, anxiety and stress, severity of low mood, cognition, gut microbiota composition, gut symptomatology, markers of immune function, gut inflammation, systemic metabolites, endothelial integrity and oxidative stress, body composition, perceived wellbeing, sleep, quality of life, resource use and cost-effectiveness.

**Discussion:**

This study will advance our understanding of the possible impact of milk proteins on psychological distress in women as well as elucidate mechanisms underpinning any association. Given dairy products form a substantial component of traditional and Western diets, the implications of these findings are likely to be of clinical and public health importance.

**Trial registration:**

The trial protocol has been prospectively registered with the Australia and New Zealand Clinical Trials Registry, ACTRN12618002023235. Registered on 17 December 2018.

**Supplementary Information:**

The online version contains supplementary material available at 10.1186/s13063-021-05812-6.

## Introduction

### Background and rationale

High prevalence mental disorders, such as depression and anxiety, are leading contributors to global disease burden and are associated with reduced quality of life and significant economic costs [1]. More generalised symptoms of psychological distress are widespread and at higher levels are estimated to affect one in eight (13%) Australians [2, 3]. As such, modifiable risk factors that may influence disease burden, such as dietary intake, have been the focus of recent research [4, 5].

Dairy products, including milk, yoghurt and cheese, are recommended as integral components of a high-quality diet and are also associated with broader health outcomes ranging from metabolic to mental disorders [6]. Recently, the role of dairy product consumption in mental disorders (i.e. depression and anxiety) has been the focus of increased research interest due to the potential of dairy products to interfere with biological pathways (e.g. inflammation) that have been implicated in these disorders [7, 8]. However, the relationship between dairy and depression has been reported inconsistently in epidemiological studies [9, 10]. For instance, milk consumption has been inversely associated with the risk of depressive symptoms in men [9], whereas milk consumption ≥ 250 mL/day of milk has been associated with an increased risk for de novo clinical depression in post-menopausal women [10]. Moreover, although studies have examined the differences in levels of dairy intake and fat composition on depression outcomes [11], none have considered the unique beta-casein profiles of dairy products, which may play a role in these differential associations. As such, the possible role of dairy in depression and anxiety, and its potential to influence biological pathways pertinent to these mental disorders, remains unclear.

Prior studies have attributed associations between the consumption of dairy products, particularly milk, and cardiovascular diseases and type 1 diabetes to a beta-casein protein variant present in milk [12, 13]. Beta-casein is a major protein component of cow’s milk, of which A1 and A2 beta-casein are two predominant types [14, 15]. Whilst A2 is thought to be the original beta-casein, a point mutation that occurred 5000–10,000 years ago in European herds produced the A1 variant [16]. The A1/A2 hypothesis was informed by these variations in amino acid sequences in beta-casein proteins and their subsequent biological effects [17]. Due to their structural variations, A1 and A2 proteins are digested differently in the gut by proteolytic enzymes; the A1 beta-casein fraction in milk produces a short opioid-like peptide, known as beta-casomorphine-7 (BCM-7), whilst the A2 beta-casein fraction yields minute quantities of BCM-7 [18].

A growing body of evidence also suggests that A1 beta-casein and BCM-7 are implicated in pathways that are believed important to the aetiology of mental disorders, including inflammation, oxidative stress and the gut microbiota [19, 20]. For example, two animal [21, 22] and three human [23–25] studies showed that administering A1 beta-casein isolates, or milk containing A1 beta-casein, increased humoral immune markers and other inflammatory markers. Concordantly, another study in humans reported that consumption of conventional milk containing A1 beta-casein was associated with reduced serum glutathione (GSH) levels compared to A2 milk [26]. Reductions in the antioxidant GSH can increase the inflammatory, oxidative and nitrosative stress burden, thus potentially exacerbating depressive symptoms [27]. In animal studies, BCM-7 increased gastrointestinal (GI) transit time via (mu) μ-opioid receptor activity [28–30]. Prolonged transit time may perturb gut microbiota composition, which is believed to be of importance to mental disorders [31–33]. Moreover, pre-clinical evidence shows that BCM-7 influences brain regions and systems implicated in neurological conditions such as schizophrenia and autism [34]. Although there is a lack of clinical evidence examining the effect of A1 beta-casein on mood symptomology in humans, significantly elevated concentrations of BCM-7 have been observed in blood plasma [35, 36] and urine [37, 38] of patients with schizophrenia, autism and postpartum psychosis.

### Specific objectives and hypothesis

Taken together, these findings suggest that A1 beta-casein consumption may have negative implications for mental health. However, a possible causal impact of A1 beta-casein consumption and symptoms of psychological distress has not been assessed in humans. Therefore, the primary aim of this randomised controlled trial is to evaluate the comparative effects of dairy products containing only A2 beta-casein, versus conventional dairy products that contain both A1 and A2 beta-casein proteins, on symptoms of psychological distress in women with low mood. This study will focus on women as we have previously shown that milk consumption is associated with an increased risk of de novo clinical depression in post-menopausal women [10]. Moreover, females experience higher rates of mood disorders compared to males [39]. The specific objectives are to:

1. Assess whether 16-week consumption of A2 beta-casein only dairy products will result in a reduction of psychological distress in women with low mood as assessed by mean change in the 21-item Depression Anxiety and Stress Scale (DASS-21) total score at the completion of intervention phase, compared to conventional A1/A2 beta-casein dairy products.
2. Assess the potential effects of 16-week consumption of A2 dairy products on symptoms of depression, anxiety and stress, severity of low mood, cognition, gut microbiota composition, gut symptomatology, markers of immune function, gut inflammation, systemic metabolites, endothelial integrity and oxidative stress, body composition, perceived wellbeing, sleep, quality of life, resource use and cost-effectiveness.

## Methods

### Trial design and setting

The Moo’D Study is a 16-week, superiority, parallel group, triple-blinded (i.e. participants, trial research team and study statistician), randomised, controlled trial conducted at the University Hospital Geelong, Australia. The study will randomise participants with 1:1 allocation ratio to either the intervention group (A2 beta-casein only dairy products) or the control group (conventional dairy products). The trial protocol has been prospectively registered with the Australia and New Zealand Clinical Trials Registry (ANZCTR; registered 17/12/18, ACTRN12618002023235) and was developed in accordance with the Standard Protocol Items: Recommendations for Interventional Trials (SPIRIT) statement [40]. The study has received ethical approval from the Barwon Health and Deakin University Human Research Ethics Committees (HREC) and will be conducted in accordance with the International Conference on Harmonisation (ICH) Guidelines for Good Clinical Practice (GCP) and will be reported using the Consolidated Standards of Reporting Trials (CONSORT) statement [41].

### Eligibility criteria and recruitment

Ninety women with low mood (score of ≥ 5 on the 8-item Patient Health Questionnaire; PHQ-8) will be recruited from the Greater Geelong and Melbourne regions. Table 1 provides the inclusion and exclusion criteria. Recruitment strategies may include a combination of online and community-based approaches such as social media posts (Facebook, Instagram, and Twitter); paid Google and Facebook advertisements; paid advertisements in local newspapers; free community talks on mental health; and displaying of flyers and pamphlets around Deakin University campuses, in places potential participants frequent (such as gyms, libraries and schools) and in clinics of general practitioners, psychologists and counsellors in the Greater Geelong region who are likely to consult with females with low mood. Study information will be listed on the study website (www.foodandmoodcentre.com/themoodstudy) and the Barwon Health Clinical Trial webpage. Trialfacts, an online recruitment service, will also be used to recruit and screen participants through social media campaigns and their existing patient database.

**Table 1:** Eligibility criteria

Inclusion criteria	Exclusion criteria
- Female - 18–75 years (at baseline) - Low mood (at baseline) as determined by a score of 5 or higher on PHQ-8 - Current conventional milk consumption of ≥ 250 ml serve/day - Willingness to commit to consuming only dairy products provided by the study - Available for intervention duration - Able to understand study materials and directions, in English - Must have access to internet and a computer/smartphone/tablet - Be willing to comply with all requirements and procedures of the study - Agree not to enrol in another interventional clinical research trial whilst part of the study	- Current consumer of A2 dairy products - Cow’s milk (dairy) allergy (established diagnosis) - Lactose intolerance (established diagnosis) - Pregnant, planning to become pregnant, or lactating - History of dementia and/or stroke - Diagnosed with or commenced new treatment for, anxiety and/or depression, within 1 month prior to baseline - GI diseases or past major GI surgery likely to interfere with study outcomes (e.g. ulcerative colitis, Crohn's disease, faecal impaction, coeliac disease, hemi colectomy, ileostomy, and colostomy) - Regular use of morphine/opioid-based medications or recreational/illicit drugs - Antibiotic use within the past month prior to baseline

*PHQ-8* Patient Health Questionnaire-8, *GI*, gastrointestinal

### Intervention and control

Participants will be randomised to receive either A2 beta-casein only dairy products (intervention) or conventional dairy products containing both A1 and A2 beta-casein proteins (control). The intervention products (A2 beta-casein only dairy products) are naturally free of the A1 beta-casein protein but are otherwise nutritionally, visually and in taste and texture, equivalent to the control products (conventional dairy products). Beta-casein levels will be measured by an independent laboratory to determine the ratio of A1 and A2 beta-casein in investigational and control products. Both the intervention and control products will be prepared and packaged by the a2 Milk Company. The packaging for products will also be identical except for printed batch codes and expiry dates. Batch codes and expiry dates will be concealed as detailed in blinding procedures below.

Participants in both the intervention and control arm will receive skim milk (ultra-heat treatment “UHT” milk) and full fat cheddar cheese (200 g block) according to their allocation (A2 or conventional dairy products). In accordance with dietary guidelines, reduced-fat products have been selected where available [42]. Participants in both the intervention and control arm will be advised to replace their usual milk and cheese with the study products and consume these in amounts they would normally consume (habitual consumption of at least 250 mL of study milk each day is an inclusion criterion). This is both to ease participant burden and enable examination of quantity of dairy consumed as a potential effect modifier. Participants in both the intervention and control arm will also be required to refrain from consuming all other cow’s milk products where protein makes a significant contribution to the overall nutritional composition, such as milk, yoghurt, cheese, kefir and mixed foods with dairy as a predominant ingredient, e.g. cream-based pasta sauces or dairy-based desserts. Otherwise, participants will be advised to maintain their usual diet. At study visit 1 (week 0), participants will receive dietary advice to aid with the selection of alternative products to replace those that are restricted within the intervention. For example, participants will be advised to consume goat’s or sheep’s yoghurt as a substitute for cow’s milk yoghurt, or to make their own yoghurt using the study products. These products are naturally free of the A1 beta-casein protein and are permitted to minimise unnecessary dietary restriction and aid with adherence to the intervention. Participants will also be advised that nutritional counselling is available throughout the study intervention should participants have difficulty adhering to the dietary intervention or if adherence results in undesirable weight changes.

### Adherence

Adherence to the dietary prescription will be measured through the completion of hard-copy daily dairy diaries. Participants will be required to record how many serves of study milk and cheese they consume each day, to the nearest ½ serve. Consistent with the Australian Dietary Guidelines, a serve of milk has been defined as 250 mL and a serve of cheese as 40 g [42]. Food models will be used to educate participants on standard serve sizes. Additionally, participants are to complete an online questionnaire each week detailing the total number of serves of milk and cheese and any additional dairy products they consumed not provided by the study. To ensure that participants comply with the intervention, answers will be checked weekly by research personnel. Participants will be instructed to return their completed dairy diaries when they collect their study products. Adherence will be cross-checked with the data entered by participants online.

### Randomisation

Following the completion of study visit 1, participants will be randomly assigned, on a 1:1 ratio, to receive either the A2 beta-casein only (A, intervention) or conventional dairy products (B, control) using stratified permuted block randomisation. Four strata will be constructed based on two variables: age (18–49, 50–75) and mood (PHQ-8 scores: 5–14; 15–24). An unblinded research assistant will use a computer-generated randomisation programme to generate a randomisation table using random block sizes, which will be partitioned by four strata based on the age and PHQ-8 scores.

### Blinding

An unblinded research assistant will prepare four blinded ‘study kit’ allocation lists, one for each stratum. A random unique three-character alphanumeric study kit code (e.g. KL5, ZA1, CJ8) will be added in place of each group (A or B). The unblinded research assistant will make up study kits according to the group and label each with the appropriate kit code. This will ensure that study investigators, study statistician and participants will be blinded to the group allocation. The unblinded research assistant will not be involved in the analysis of study findings to preserve study blinding. Group allocations will only be available once all data collected have been entered into the study database for every participant and the database has been locked. Unblinding of intervention groups will only occur once statistical analysis is completed, except in the case of an emergency.

### Implementation

Participants will be enrolled at study visit 1 by a blinded research assistant or investigator. After the completion of study visit 1, participants will be randomised by a blinded researcher and allocated a study kit code on a first-in-first-out assignment from the appropriate blinded ‘study kit’ allocation list depending on participant age and mood. A blinded research assistant or investigator will dispense study kits to participants according to the allocated study kit code. At each dairy collection visit, blinded researchers will dispense pre-packaged study kits to participants according to the allocated study kit code.

### Participant timeline

**Fig. 1:**
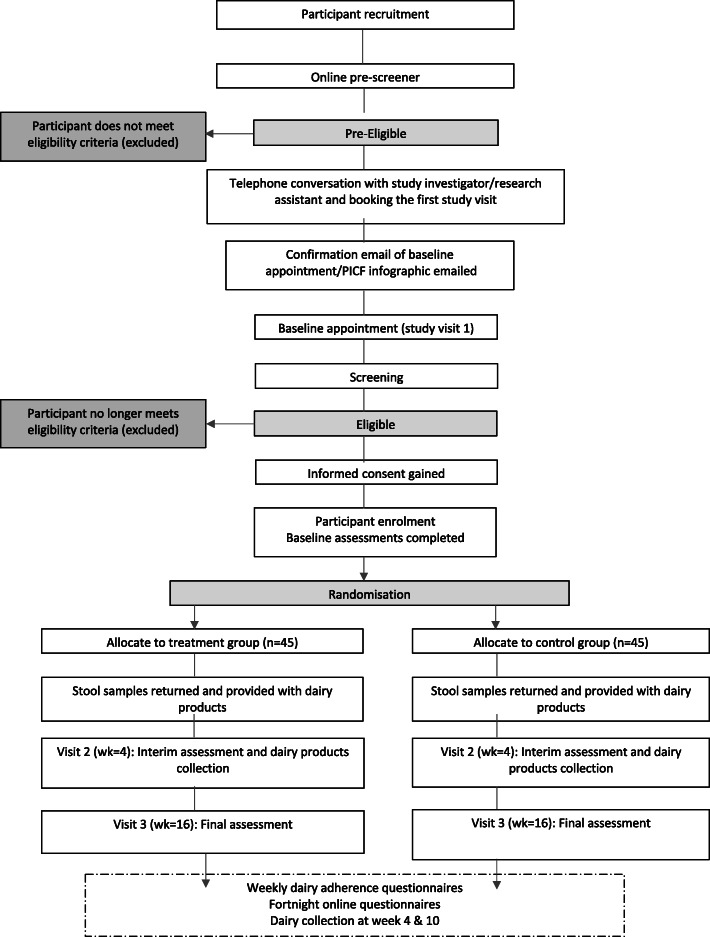
Study flow diagram detailing study procedures for The Moo’D Study

Figure 1 provides a schematic diagram of enrolment, interventions and assessments. Participants will complete three study visits (weeks 0, 4, 16) in addition to completing fortnightly assessments online (see Table 2). Participants will also collect their dairy products at two of these visits (weeks 0 and 4) and return to the study centre to collect additional products at week 10.

**Table 2:** Schedule of assessments and instruments implemented at each study timepoint

	Enrolment	Allocation	Study period (weeks)								
Timepoint (weeks)	0	0	0^**c**^	2	4^**c**^	6	8	10	12	14	16^**c**^
**ENROLMENT**											
Online pre-screening	✓										
Eligibility screen	✓										
Informed consent	✓										
Randomisation		✓									
**INTERVENTIONS**											
A2 beta-casein only dairy (intervention)			✓	✓	✓	✓	✓	✓	✓	✓	✓
A1/A2 beta-casein dairy (control)			✓	✓	✓	✓	✓	✓	✓	✓	✓
**ASSESSMENTS (INSTRUMENT)**											
Psychological distress (DASS-21)			✓	✓	✓	✓	✓	✓	✓	✓	✓
Severity of depressive symptoms **(**PHQ-8)			✓^a^	✓	✓	✓	✓	✓	✓	✓	✓
Gastrointestinal symptoms (VAS-IBS)			✓	✓	✓	✓	✓	✓	✓	✓	✓
Stool formation (BSFS)			✓	✓	✓	✓	✓	✓	✓	✓	✓
Functional gastrointestinal disorders (ROME IV module)			✓								✓
Cognitive assessments (Cogstate)			✓								✓
Self-reported health/adverse effects				✓	✓	✓	✓	✓	✓	✓	✓
Perceived wellbeing (WHO-5)			✓								✓
Quality of life (AQoL-8D)			✓								✓
Self-reported health care utilisation (Resource use questionnaire)			✓								✓
Sleep (Athens Insomnia Scale)			✓								✓
Dietary intake (DQES v3.2)			✓								✓
Physical activity (IPAQ-SF)			✓								✓
Personality disorders screener (SAPAS)			✓								
Demographics (self-report)			✓								
Medications and dietary supplements (self-report)			✓								✓
Medical conditions and history (self-report)			✓								✓
Anthropometrics (stadiometer, weighing scales, measuring tape)			✓								✓
Body composition (DXA)			✓								✓
Gut microbiota (faecal, 16s RNA faecal analysis, metagenomics^b)^			✓		✓						✓
Markers of immune functions (blood, mesoscale discovery platform)			✓		✓						✓
Metabolomics – BCM-7 (blood, HPLC coupled to MS)			✓		✓						✓
Metabolomics – SCFA (faecal, GC-MS^b^ or HPLC analysis of faeces)			✓		✓						✓
Markers of oxidative stress (blood, glutathione assay kits)			✓		✓						✓

^a^Also completed at online pre-screening prior to preliminary baseline assessments

^b^Dependent on further funding

^c^In-clinic assessment

*AQoL-8D* Assessment of Quality of Life 8D, *BSFS* Bristol Stool Form Scale, *DASS-21* Depression Anxiety Stress Scale 21 items, *DQES* Dietary Questionnaire for Epidemiological Studies, *DXA* dual-energy X-ray absorptiometry, *HPLC* high-performance liquid chromatography, *IPAQ-SF* International Physical Activity Questionnaire short form, *MS* mass spectrometry, *GC-MS* gas chromatography-mass spectrometry, *PHQ-8* Patient Health Questionnaire 8-items, *SAPAS* Standardised Assessment of Personality Abbreviated Scale, *VAS-IBS* Visual Analogue Scale for Irritable Bowel Syndrome, *WHO-5* World Health Organization Wellbeing Index 5-items

#### Pre-screening

Potential participants will complete an online screening questionnaire. If eligible and willing to participate, they will be contacted by the study team to book their first study visit.

#### Study visit 1 (week 0)

At the commencement of study visit 1, participants will be re-screened against the eligibility criteria. If eligible, research personnel will ask participants to provide informed consent and will enrol participants in the study before the collection of any baseline measures. During this visit, participants will receive advice regarding the diet intervention and be provided with instructions on dairy storage, a hard-copy dairy diary to record their daily intake and a stool sample collection kit. Participants will also complete their baseline assessments, which include the collection of questionnaire data, a cognitive assessment, blood collection and a body composition scan (Table 2). On completion of study visit 1, participants will be randomised to either the intervention or control group. Participants will be required to return to the study centre within 72 h of their first study visit to collect their dairy products and return their completed stool sample.

#### Intervention period

Within 2 weeks of commencing the intervention, a study member will contact participants by phone to discuss adherence, trouble-shoot any difficulties encountered during the study period and book additional study visits. In between clinical assessments, participants will complete online fortnightly self-report questionnaires and a weekly adherence questionnaire (see Table 2). The final (week 16) self-report questionnaires will be emailed to participants to complete online, to reduce burden and minimise time spent in-clinic.

#### Study visit 2 (week 4)

Participants will return their completed stool sample, provide a blood sample and collect additional study products.

#### Dairy collection visit (week 10)

Participants will return to the study centre to collect additional study products.

#### Study visit 3 (week 16)

Participants will return their completed stool sample and provide a blood sample. Participants will also complete a final cognitive assessment and body composition scan.

### Primary outcome measure

The primary outcome is between-group differences from baseline to week 16 in symptoms of psychological distress, measured by total scores summed from the 21-item self-reported Depression Anxiety and Stress Scale (DASS-21). Questions on this scale pertain to feelings over the past week; however, to align with study assessment periods, participants will be required to rate how they were feeling in the past 2 weeks. Responses to each item are made on a 4-point scale from 0 (did not apply to me at all) to 3 (applied to me very much or most of the time), producing a maximum score of 63. Higher scores indicate more symptoms of dysphoric mood, whilst a score of 0 indicates the absence of disturbed mood symptoms rather than a positive mood state. Previous studies have demonstrated good validity and also high internal consistencies for all three subscales of the DASS-21 [43, 44].

### Secondary outcome measures

Subscales of the DASS-21 (symptoms of depression, anxiety, stress individually) will be secondary outcomes. The 8-item Patient Health Questionnaire (PHQ-8) will be used as a screening measure to screen for low mood and will be administered fortnightly to measure changes in the severity of low mood. The PHQ-8 will also be used to measure changes in mood to detect any untoward changes in the severity of low mood throughout participants’ involvement in the trial.

Cognitive performance will be measured by a validated computerised cognitive brief battery, which has been used extensively in clinical trials, developed by Cogstate (Cogstate Ltd., Melbourne, Australia). The four tasks comprising the Cogstate Brief battery, from which main outcome variables will be analysed: Working Memory (One Back); Visual Learning (One Card); Psychomotor Function (Detection); and Attention (Identification). Additionally, the Paired Associate Learning (Continuous Paired Associate Learning) will be used as a measure of memory processes relating to encoding and retrieval [45, 46]. Both measures have acceptable construct and criterion validity in a neuropsychological context [47].

The World Health Organization (WHO) WellBeing Index will be utilised to assess perceived wellbeing. Each statement is rated on a Likert scale (0–5) to assess how applicable they are to the participant’s feelings in the past 14 days. The total score indicates the level of wellbeing, which may range from an absence to maximal wellbeing [48].

The Athens Insomnia Scale will be utilised to assess sleep difficulty, quality and duration in the past month. It is an eight-item self-assessed psychometric instrument based on the WHO’s International Statistical Classification of Diseases and Related Health Problems (ICD-10) criteria [49].

Psychosocial aspects of quality of life will be assessed utilising the Assessment of Quality of Life (AQoL)-8D, a Multi-Attribute Utility instrument developed by Monash University [49]. The instrument contains 35 items, which load onto eight dimensions. Three of these are related to a physical super-dimension (independent living, pain, senses) and the remaining five to a psychosocial super-dimension (mental health, happiness, coping, relationships and self-worth). The 35 items may be reduced to a single utility score using the AQoL-8D algorithm, to reflect health-related wellbeing [49]. The utility values will also be used to calculate quality-adjusted life years (QALYs), a standard outcome measure in economic evaluations.

To determine participants’ broader use of healthcare services, a Resource Use Questionnaire (RUQ) will be completed at weeks 0 and 16. An economic evaluation will be undertaken as a cost consequences analysis from a primarily health sector perspective with an additional partial societal perspective included. The evaluation will measure and value the use of health care resources over the trial period for those in the intervention and control groups, and then compare any additional costs to the primary and secondary outcomes including QALYs. Medical resources collected through the RUQ will be costed using standard Australian unit costs (i.e. Medicare Benefits Schedule and Pharmaceutical Benefits Scheme). Lost work productivity, also captured in the RUQ, will be costed using the human capital approach by applying an average hourly wage rate plus 25% overhead costs [50, 51].

Functional GI disorders (e.g. irritable bowel syndrome, constipation, diarrhoea, and abdominal pain) will be assessed using the Rome IV Bowel Disorders and Central Nervous System Disorders of Gastrointestinal Pain Module, a sub-module of The Rome IV Diagnostic Questionnaire for Functional Gastrointestinal Disorders in Adults (R4DQ). This module contains questions from the R4DQ (Q: 40-67) and will be used to assess GI disorders in participants [52]. The Visual Analogue Scale for Irritable Bowel Syndrome (VAS-IBS) questionnaire is a valid self-imaging tool that measure changes in GI symptoms in patients with IBS [53]. This tool will be used to capture changes in gut symptomology (e.g. bloating, abdominal pain) in the intervention and control groups every fortnight. Participant’s stool formation will be assessed using the Bristol Stool Form Scale (BSFS) questionnaire every fortnight [54].

The gut microbiota composition will be assessed via stool samples using 16S rRNA gene sequencing. This technique is extensively used in identification of bacterial taxa in both animal and human faecal samples [55]. After sequencing, the 16S rRNA gene sequences will be matched to reference databases to identify operational taxonomic units corresponding to the genus or species level. In addition, subject to funding, stool samples may be sent for metagenomics analysis, an advanced and more in-depth analysis technique for assessing microbiota composition and inferred function [56].

Biomarkers of immune function and oxidative stress will be assessed via blood samples collected from participants. Markers of immune function (tumour necrosis factor-α, interleukin (IL)-1β, IL-6, IL-10) and gut inflammation (IL-4, humoral immune markers: immunoglobulin E, immunoglobulin G) will be analysed [57, 58]. Markers of endothelial integrity such as liposaccharide-binding protein (LBP) and CD14, which are indicators of microbial product dissemination to the peripheral circulation, will also be assessed [59, 60]. Changes in oxidative stress levels will be assessed using GSH, a marker of cellular redox balance [61]. Moreover, changes in metabolomic markers such as short-chain fatty acids (SCFA) and BCM-7 will be assessed. SCFA are metabolic by-products of microbial fermentation of dietary fibre in the gut and serve as markers of colonic health [62]. This study will measure SCFA concentrations in stool samples collected from participants, subject to funding. BCM-7 that are yielded by the enzymatic digestion of A1 beta-casein protein in milk will be measured in blood samples [61].

A whole body scan will be carried out using the dual energy X-ray absorptiometry (DXA, Lunar Prodigy Pro) to study body composition, which indicates fat, lean and bone mass of the participants [63].

Data will also be collected related to participant demographics, habitual diet using the Cancer Council’s Dietary Questionnaire for Epidemiological Studies v3.2 (DQES) [64], physical activity using the short form of the International Physical Activity Questionnaire (IPAQ) [65] and symptoms of personality disorders using the Standardised Assessment of Personality Abbreviated Scale (SAPAS) [66]. These variables will be examined as potential effect modifiers in exploratory post hoc subgroup analyses.

### Adverse events

Adverse events (AE) will be monitored and recorded at all timepoints, which will be overseen by the study management team. Any unfavourable or unintended medical occurrence (e.g. sign, symptom, syndrome and illness) that develops or worsens during the trial will be reported as an AE. All AEs will be assessed for severity (mild, moderate, severe), causality (not related, unlikely, possibly, probably or definitely related to study treatment) and seriousness and will be documented by the study team. For any AE (regardless of seriousness or severity), researchers will maintain contact with participants until it has been resolved and symptoms disappear. If a participant experiences a significant AE, they may be withdrawn from the study. The decision will rest with the principal investigator and the participant will be referred to their treating doctor, if required. In the case of a serious adverse event (SAE), the HREC will be notified within 24 h of the researchers becoming aware of the event. Emergency unblinding will occur for an SAE deemed related to the study product.

### Power calculation

To date, no studies have investigated the effects of A2 beta-casein only vs conventional dairy products on symptoms of psychological distress. For this reason, the anticipated effect size is largely based on findings derived from the broader nutrition literature [67–71]. A feasible sample size of 80 participants (40 per study arm) has been calculated to detect a medium intervention effect size of 0.63 (standard mean differences) defined as a between-group standard mean difference in DASS-21 total score from baseline to 16 weeks between the intervention and control groups. We assume the standard deviation of DASS-21 score change is 3.6, which is similar to published RCTs [67–69]. A sample size of 80 participants, achieves 80% power for a two-tailed analysis with a type I error level of 0.05 to detect a between-group mean difference of 2.3 or greater in DASS-21 total score from baseline to 16 weeks between the intervention and control groups. The total sample size will be inflated to 90 to account for an anticipated attrition rate of 12.5%. If our attrition rate is higher than anticipated (e.g. due to COVID-19 pandemic), we will aim to recruit further participants.

### Statistical methods

All randomised participants with valid 0 and 16-week observations will be included in the primary analysis (modified intent-to-treat analysis). The primary outcome will be analysed using generalised estimating equation (GEE), using an identity link function and normal distribution, to test the between-group differential change in DASS-21 total score from baseline to 16 weeks follow-up between the intervention and control groups. This will be estimated through two-way interaction between group allocation and follow-up time points in a GEE model that includes nominal intervention allocation factor, nominal measurement time and the two-way interaction between allocation and measurement time. In order to account for within-participants’ autocorrelation due to repeated measurements, Huber/White cluster sandwich variance estimates will be used to account for clustering from the same study participants [72]. As a secondary analysis, an additional GEE model will be implemented that includes all observations with at least one post-baseline measure. All other secondary longitudinal continuous outcome analyses will follow the same method as the primary outcome analysis. Sensitivity analysis may be used to evaluate missing at random assumptions for missing follow-up data. This will be done by imputing data assuming missingness is positively or negatively associated with outcomes in the intervention and control group and then the intervention effect sizes will be estimated from these different imputation scenarios. An additional sensitivity analysis will be performed with a GEE model adjusted for baseline measurements as potential covariates. Exploratory post hoc subgroup analyses may also be conducted if feasible to explore possible moderation of intervention effects (e.g. menopause status). Cross-sectional associations of baseline demographic and prognostic variables (e.g. habitual diet, exercise, personality, biomarkers) will also be explored. This work will be independent to the main trial report and will be conducted subject to further funding and staff resourcing.

### Data management, monitoring and dissemination

This research is led by investigators at Deakin University and is sponsored by Deakin University. The study sponsor is responsible for approving protocol amendments when ethics approvals have been obtained. In line with the ethical approval for this study, all data will be stored (re-identifiable) in a locked cabinet with secure-swipe card access, and on secure servers housed by Deakin University and Barwon Health. Access will be restricted to members of the study team only. All enrolled participants will be provided with a unique study ID number to protect the confidentiality of participants. Data and documentation relating to the study will be retained for at least 15 years. A data monitoring committee was not assembled due to the low-risk nature of the intervention and trial methods. On completion of the trial, participants will receive a report outlining the key study findings. Study investigators will also disseminate findings, presented as aggregate data only in the form of journal articles, conference presentations, seminars and community presentations. The funding body, a2 Milk Company, had no input into the design or conduct of the study and approval to publish the results of the study is not required.

## Discussion

The existing body of evidence concerning dairy consumption and depression is inconsistent and equivocal, and lacks data on putative mechanistic pathways to explain previously observed outcomes [11]. By employing a rigorous triple-blinded, RCT design, this study will be the first to evaluate possible differential effects of A2 beta-casein only vs conventional dairy product consumption on symptoms of psychological distress. Through exploration of a range of secondary health outcomes, findings from this study will also expand our understanding of the biological effects of A1 and A2 beta-casein consumption in women and may provide novel insights into possible differential health effects of these dairy products.

A significant challenge with many dietary trials is blinding of the study intervention, which is important to minimise expectation bias [73]. A strength of this study is its triple-blind design; participants, the study statistician, researchers conducting study assessments and analysing study outcomes will be blinded to group allocation until completion of study analyses. Further, this study will have real-world applicability given that participants will consume dairy products in line with their usual intake and a range of A2 beta-casein products are widely available in mainstream food and grocery settings. Consideration has also been given to intervention duration; prior studies have indicated that dietary changes can affect depressive symptoms in as little as 3 weeks [71]; thus, the duration of the intervention (16 weeks) should be long enough for us to adequately examine the effects of beta-casein consumption on symptoms of psychological distress.

To summarise, this study will be the first to evaluate the possible differential effects of A2 beta-casein only vs conventional dairy consumption on symptoms of psychological distress. It will also expand our understanding of the biological effects of A1 and A2 beta-casein consumption and may provide novel insights into possible differential health effects of these dairy products in women with low mood. Findings from this study may have implications for the food industry, consumers and policy makers, particularly given the widespread consumption of dairy products. Further, outcomes from this study may empower consumers with knowledge to make informed choices in regard to selecting dairy products for consumption. This study will add to the growing evidence base in the field of Nutritional Psychiatry and facilitate our understanding of the possible role of milk-derived beta-casein proteins in human health.

## Trial status

Recruitment for the RCT commenced in January 2019 and data collection was due to be completed by December 2020. The trial was halted in March 2020 due to social distancing regulations in response to the COVID-19 global pandemic. The trial team plans to resume recruitment upon easing of COVID-19-related restrictions in 2021. Amendments to the protocol may be employed to minimise face-to-face contact with trial participants. Any amendments will be updated on the ANZCTR.

## Protocol amendments

The trial Protocol is version 13 date 16/12/2019. The research ethics boards have approved changes to the protocol since inception of the trial. All changes have been updated in ANZCTR and communicated to relevant parties. Any further protocol amendments will be communicated with the ethics committee and any parties affected.

## Supplementary Information

**Additional file 1..**

**Additional file 2..**

## Data Availability

Data sharing does not apply to this manuscript as it is a protocol of an ongoing trial and no data are reported. Only trial investigators, who have been approved by ethics, will have access to the final trial dataset.

## Abbreviations

ANZCTR: Australia and New Zealand Clinical Trial Registry
AQoL: Assessment of Quality of Life
AE: Adverse events
BCM-7: Beta-casomorphine-7
BMI: Body mass index
BSFS: Bristol Stool Form Scale
CONSORT: Consolidated Standards of Reporting Trials
DASS-21: 21-item Depression Anxiety and Stress Scale
DQES: Dietary Questionnaire for Epidemiological Studies
DXA: Dual energy X-ray absorptiometry
GCP: Guidelines for Good Clinical Practice
GEE: Generalised estimating equation
GI: Gastrointestinal
HREC: Human Research Ethics Committees
ICD: International Statistical Classification of Diseases and Related Health Problems
IL: Iinterleukin
LBP: Liposaccharide-binding protein
PHQ-8: 8-Item Patient Health Questionnaire
QALYs: Quality-adjusted life years
RCT: Randomised controlled trial
RUQ: Resource Use Questionnaire
R4DQ: The Rome IV Diagnostic Questionnaire for Functional Gastrointestinal Disorders in Adults
SAE: Serious adverse event
SAPAS: Standardised Assessment of Personality Abbreviated Scale
SCFA: Short-chain fatty acids
UHT: Ultra-heat treatment
VAS-IBS: The Visual Analogue Scale for Irritable Bowel Syndrome
WHO: World Health Organization
