# Supplementary material for: The Moo’D Study: protocol for a randomised controlled trial of A2 beta-casein only versus conventional dairy products in women with low mood
Source: Trials. 2021 Dec 11;22:899. doi: 10.1186/s13063-021-05812-6 (PMC8665310; doi:10.1186/s13063-021-05812-6)
Supplement: Supplementary file 1 — Additional file 1.. [file 13063_2021_5812_MOESM1_ESM.docx]

**Additional file 2:** Items from the World Health Organization Trial Registration Data Set

| **Item** | **Description** |
| --- | --- |
| 1. Primary registry and trial-identifying number | Australia and New Zealand Clinical Trials Registry, ACTRN12618002023235. |
| 2. Date of registration in primary registry | Registered on 17 December 2018 |
| 3. Secondary identifying numbers | n/a |
| 4. Sources of monetary or material support | The a2 Milk Company have provided unrestricted funding for this project. The donors will also provide, in-kind, the investigational products (intervention and control) for the duration of the study and analysis of selected biomarkers by an independent laboratory. The donors play no role in study design, collection, analysis, or reporting of the results. Donor: The a2 Milk Company Limited – NZ Co. No. 1014105, NZBN: 9429037368845 |
| 5. Primary Sponsor | Deakin University |
| 6. Secondary Sponsor | n/a |
| 7. Contact for Public Queries | Professor Felice Jacka ; felicejacka@gmail.com |
| 8. Contact for Scientific Queries | Professor Felice Jacka; felicejacka@gmail.com |
| 9. Public title | The Moo’D Study |
| 10. Scientific title | The Moo’D Study: Protocol for a randomised controlled trial of A2 beta-casein only versus conventional dairy products in women with low mood |
| 11. Countries of recruitment | Australia |
| 12. Health condition(s) or problem(s) studied | Psychological distress |
| 13. Intervention(s) | A2 beta-casein only dairy products (intervention) vs conventional dairy products containing both A1 and A2 beta-casein proteins (control). |
| 14. Key inclusion and exclusion criteria | Inclusion criteria:   - Female - 18-75 years (at baseline) - Low mood (at baseline) as determined by a score of 5 or higher on the Patient Health Questionnaire-8 (PHQ-8) (Appendix 3) - Current conventional milk consumption of ≥250ml serve/day - Willingness to commit to consuming only dairy products provided by the study - Available for intervention duration - Able to understand study materials and directions, in English - Must have access to internet and a computer/smartphone/tablet - Be willing to comply with all requirements and procedures of the study - Agree not to enrol in another interventional clinical research trial while part of the study   Exclusion criteria:   - Current consumer of A2 dairy products - Cow’s milk (dairy) allergy (established diagnosis) - Lactose intolerance (established diagnosis) - Pregnant, planning to become pregnant, or lactating - History of dementia and/or stroke - Diagnosed with or commenced new treatment for, anxiety and/or depression, within 1 month prior to baseline - Gastrointestinal (GI) diseases or past major GI surgery likely to interfere with study outcomes (e.g. ulcerative colitis, Crohn's disease, faecal impaction, coeliac disease, hemi colectomy, ileostomy, and colostomy) - Regular use of the following:   - morphine/opioid-based medications   - recreational/illicit drugs - Antibiotic use within the past month prior to baseline |
| 15. Study type | 16-week, superiority, parallel group, triple-blinded (i.e., participants, trial research team and study statistician), randomised, controlled trial |
| 16. Date of First Enrolment | 22^nd^ January 2019 |
| 17. Target Sample Size | 90 participants |
| 18. Recruitment Status | Enrolling |
| 19 Primary Outcome | Symptoms of psychological distress |
| 20. Key Secondary Outcomes(s)  Clinical Outcomes | Symptoms of depression, anxiety and stress, severity of low mood, cognition, gut microbiota composition, gut symptomatology, markers of immune function, gut inflammation, systemic metabolites, endothelial integrity and oxidative stress, body composition, perceived well-being, sleep, quality of life, resource use and cost-effectiveness. |
