# Supplementary material for: The Moo’D Study: protocol for a randomised controlled trial of A2 beta-casein only versus conventional dairy products in women with low mood
Source: Trials. 2021 Dec 11;22:899. doi: 10.1186/s13063-021-05812-6 (PMC8665310; doi:10.1186/s13063-021-05812-6)
Supplement: Supplementary file 2 — Additional file 2.. [file 13063_2021_5812_MOESM2_ESM.pdf]

DEAKIN UNIVERSITY

GIFT AGREEMENT

The a2 Milk Company Limited

This a2 Milk Company Ltd / Deakin Collaboration wishes to support research at the Food & Mood Centre within IMPACT Strategic Research Centre, Deakin University.

PURPOSE OF THE GIFT

The a2 Milk Company Limited (NZ Co. No. 1014105) a limited liability company, with its registered office at Level 10, 51 Shortland Street, Auckland 1010, New Zealand ("the Donor") shall give Deakin University (ABN 56 721 584 203) ("the University") the total sum of up to $400,000 as set out in this gift agreement for the purpose of funding research at the Food & Mood Centre within IMPACT Strategic Research Centre at Deakin University.

- Total gift of up to AUD $400,000 over two years.
- The funding will provide the opportunity for research to develop the evidence base concerning associations between dairy consumption and mental, brain and gut health in humans ("the Research").

Commitment
The University is committed to ensuring this gift is used for its intended purpose (being to fund the Research). In the event that any part of the gift that has already been paid by the Donor to the University, for any reason, cannot be used for the purpose here intended, the University will endeavour, where possible in consultation with the Donor, to use the gift for a purpose that will accomplish the Donor's wishes or is consonant with the spirit and intent of the Donor's gift.

RECOGNITION

The a2 Milk Company will be recognised as a donor to the Deakin Food and Mood Centre and its support of research at the Food and Mood Centre will be acknowledged in public materials, including research reports and publications.

The University strives to develop long-term relationships with all donors. The University uses a variety of methods to recognise major gifts both within the University and in the public domain.

University staff will give the Donor an overview of these methods and suggest an appropriate way to recognise your gift. The Donor can either then approve the suggested recognition activities or indicate that they are not required.

If the recognition activities include internal and/or external publicity or news announcements, these will only be distributed with the Donor's permission.

The Donor and the University each agree that they will only use the other's logo and trade marks if it has the other's written consent and then only in accordance with the express instructions given by the other from time to time.

REPORTING

A progress report outlining the impact of the gift will be provided at least annually for the life of the gift and in any event prior to the Donor making the second payment (in the amount of $200,000) and the third payment (in the amount of $100,000).

The Food and Mood Centre will be required to write a yearly statement about the impact of the grant.

The Donor and the University will comply with the *Privacy and Data Protection Act 2014 (Vic).*

PAYMENT

The Donor pledges to pay up to AUD *$400,000* over two years as follows:

- $100,000 on or about 13 April 2018
- $200,000 on or before 31 July 2018, provided that the Research has commenced, is ongoing and that the Donor (acting reasonably and having regard to the progress report(s) provided by the University) is satisfied that the gift is being used for its intended purpose
- $100,000 on or before 31 July 2019, provided that the Research has commenced, is ongoing and that the Donor (acting reasonably and having regard to the progress report(s) provided by the University) is satisfied that the gift is being used for its intended purpose

Fund name and number: The a2 Milk Company – Research Project

In addition to the progress report referred to above, the University will send a pledge reminder one month in advance of the dates listed above, including details of payment options.

CORRESPONDENCE

The University will send correspondence relating to this gift to:
Andrew Clarke, Chief Scientific Officer,
The a2 Milk Company Limited
Level 10, 51 Shortland Street, Auckland 1010, New Zealand

The Donor should send correspondence to:
Deakin University
Central Gifts Management
Advancement
Melbourne Burwood Campus
221 Burwood Highway
BURWOOD VIC 3125

SIGNED BY

....<br>Geoffrey Babidge<br>Managing Director and CEO<br>The a2 Milk Company Limited<br><br>Date: 12/04/18	....<br>Ron Fairchild<br>Vice-President and Chief Advancement Officer<br>Deakin University<br><br>Date: 17/04/18
